# Inflammation and autoimmune myasthenia gravis

**DOI:** 10.3389/fimmu.2023.1110499

**Published:** 2023-01-30

**Authors:** Ruksana Huda

**Affiliations:** Department of Microbiology and Immunology, University of Texas Medical Branch, Galveston, TX, United States

**Keywords:** myasthenia gravis, inflammation, autoimmune diseases, AChR, autoantibody, IL-6

## Abstract

Myasthenia gravis (MG) is a neuromuscular autoimmune disorder characterized by chronic but intermittent fatigue of the eye- and general body muscles. Muscle weakness is caused primarily by the binding of an autoantibody to the acetylcholine receptors, resulting in blockage of normal neuromuscular signal transmission. Studies revealed substantial contributions of different proinflammatory or inflammatory mediators in the pathogenesis of MG. Despite these findings, compared to therapeutic approaches that target autoantibody and complements, only a few therapeutics against key inflammatory molecules have been designed or tested in MG clinical trials. Recent research focuses largely on identifying unknown molecular pathways and novel targets involved in inflammation associated with MG. A well-designed combination or adjunct treatment utilizing one or more selective and validated promising biomarkers of inflammation as a component of targeted therapy may yield better treatment outcomes. This review briefly discusses some preclinical and clinical findings of inflammation associated with MG and current therapy approaches and suggest the potential of targeting important inflammatory marker(s) along with current monoclonal antibody or antibody fragment based targeted therapies directed to a variety of cell surface receptors.

## 1 Introduction

Myasthenia gravis (MG) is an autoimmune disorder characterized by muscle weakness and fatigue associated with autoantibodies binding to acetylcholine receptors (AChRs) distributed at the neuromuscular endplates (1). Autoantibody (IgG1 and IgG3 isotypes) binding reduces the availability of functional acetylcholine receptors (AChRs) to Acetylcholine through receptor blockade and internalization. Receptor depletion is further intensified through myocyte destructions by activated complement and membrane attack complexes (2, 3). About 40% of AChR autoantibody-seronegative patients showed the presence of either muscle-specific tyrosine kinase (MuSK) (predominantly IgG4 isotype) or LDL receptor-related protein (LRP)4 (complement-activating IgG1 isotype) -specific autoantibodies that were known to disperse postsynaptic AChR clusters (4, 5) to cause AChR deficiencies.

Inflammation has long been regarded as an important contributing factor for both generalized and ocular MG pathogenesis, occurring in about 80% and 50% of MG patients, respectively (6, 7). The mediators of inflammation, predominantly cytokines and chemokines are released mostly by inflammatory monocytes and macrophages in the affected postsynaptic neuromuscular junction (NMJ) or thymic tissue (8–10), which then move also into the peripheral circulation. Subsequently, AChR-specific T-helper cells infiltrate the NMJ or thymic microenvironment and interact with B cells to induce its autoimmune activation (11). In addition to the thymus, germinal centers (consisting mainly of B lymphocytes, T follicular helper cells (Tfh), and plasma cells) are also prominently formed in the secondary lymphoid organs, such as the spleen in experimental mouse model of MG (EAMG), to produce MG-specific autoantibody (12, 13). These cytokines directly or indirectly take part in priming and activating dendritic cells, AChR-specific T-helper cells, and other cells that orchestrate to promote maturation, proliferation, and differentiation of AChR-specific B cells into plasma cells for autoimmune development (14). Depending on the specificity of agonists and Toll-like receptors (TLRs) 3, 7, or 9 involved, several cytokines (e.g., Tumor necrosis factor/TNF) function in an autocrine or paracrine manner, controlled by the specific intracellular signaling pathways and predominantly by nuclear factor kappa B (NFκB), signal transducer and activators of transcription (STAT) and CAAT Enhancer Binding Protein (C/EBP) family of transcription factors (15). For example, IL-6 and IFN-γ are the STAT activator, whereas TNFα is an NFκB activator.

Increasing evidence shows that dysregulated inflammatory response is closely associated not only with MG but also with many autoimmune diseases, including rheumatoid arthritis (RA), systemic lupus erythematosus (SLE), multiple sclerosis (MS), etc. (14). Recent RNA-seq analysis of peripheral blood mononuclear cells (PBMCs) from AChR-MG patients (16) and our nCounter transcriptome analysis of genes modulated by epigenetic inhibition of histone deacetylases in experimental mouse model of MG revealed occurrence of significant dysregulation of inflammatory molecules in association with altered autoantibody levels (17). Further, correlative evidence from such preclinical and clinical studies demonstrates that autoantibodies can also promote or suppress inflammation based on their immunoglobulin (Ig) isotypes and Fc domain interactions with other cells (18). The mechanisms include Fc receptor (FcR) binding of immune cells and their activations, complement-fixing, and immune complex deposition resulting in functional receptor depletion.

Traditional treatments such as plasma exchange, Thymectomy, and immunosuppressive drugs do not cure MG. Current targeted therapy using monoclonal antibodies and antibody fragments against various popular biomarkers demonstrated a variable degree of effectiveness (7). However, these monotherapies neither reduced expected levels of autoantibody nor showed significant clinical improvement or disease remission. Additionally, a small fraction of patients is refractory, exhibiting a relatively low response to targeted therapy (19). Therefore, in addition to the monoclonal antibody (mAb)-based therapies against conventional immune cell-associated biomarkers, there is also a need for adjunct therapies directed to new, precise, and reliable targets for inflammation that can ensure fulfilling the current gap in targeted therapy. Thus, finding and utilizing validated promising biomarkers for combination treatment might be critical for a highly effective treatment for MG.

## 2 Role of infection and inflammation in MG etiology

Chronic inflammation in the thymus due to persistent microbial infection has been reported to occur in MG patients (20). Disruption of tolerance and generation of autoreactive cells in the thymus are seemingly the first step for AChR-MG pathogenesis. The changes are thought to be prompted by innate immune activation (e.g., TLRs) triggered in a host following a viral infection that leads to the production of an array of inflammatory molecules. Further supporting the reports, infections with certain microbes and viruses (including recent SARS-CoV-2) were found to initiate or exacerbate an autoimmune condition, likely through induction of interferon (IFN)-mediated immune response (21–24). Also, the antigenicity of exogenous molecules (e.g., a microbial protein) with significant homology with a host protein or host-derived endogenous protein associated with cell stress or cell damage is believed to act as an autoantigen to trigger such onset of the autoimmune response (25). A recent study of single-cell RNA sequencing analysis detected viral transcripts for Epstein-Barr virus and herpesvirus 6A in the thymoma of MG patients; however, the correlation with the virus was non-significant (26). Thus, the exact mechanism underlying the steps of immune activation in the thymus, contributed by infection and inflammation, is incompletely understood.

## 3 Inflammation and immune dysregulation in promoting autoimmune MG

Inflammatory mediators, consisting mainly of interleukins, interferons, and chemokines from leukocytes, play a major role in immune modulation. Studies have shown the presence of infiltrated macrophages and monocytes at the neuromuscular junction in MG (27, 28). Local release of cytokines from these cells further recruits polymorpho neutrophils (PMN) and other immune cells that permeate into the specific site and start the inflammatory cascades for immune activation. This is supported by the findings of neuromuscular-related antigen expressions (including AChRs), the presence of germinal centers (GCs), and increased frequency of Tfh cells in the thymus, altered microRNAs, and IFN signaling in subpopulations of thymic epithelial cells in MG patients with thymoma (29, 30). Importantly, in addition to B cells for the autoantibody-producing prime role, subsets of T-lymphocytes (T helper cells) also play a crucial role in launching and maintaining chronic inflammation and enhancing the magnitude of inflammatory signals to influence class switching, somatic hypermutation, and differentiation of those B cells to generate autoantibody-producing plasma cells, leading to subsequent development and progression of MG disease (31).

Recent RNA-seq analysis of peripheral blood mononuclear cells (PBMCs) from 19 AChR-MG patients with a disease onset under the age of 50 years (AChR-EAMG) reported transcriptional dysregulation of inflammatory biomarkers (32). The inflammatory proteins important in this study were IL-4, NFκB2, IFN, and RelB, among others. Dysregulation of the molecules was also correlated with the altered AChR-specific antibody levels and microRNAs. Additional studies may dissect the mechanisms underlying the findings.

Our recent nCounter transcriptome results are also in line with literature showing a key role of an inflammatory signature in the pathogenesis of a mouse model of MG (17). We demonstrated an epigenetic influence in regulating expression levels of a key inflammatory cytokine, IL-6, from AChR-stimulated PBMCs in mouse model of MG. We further identified altered expression of many previously unknown genes associated with IL-6 suppression, which was further correlated with autoantibody reduction, by inhibiting an epigenetic molecule, histone deacetylase-2. Future directions in this field should include a comprehensive analysis of individual genes through gene deletion, and bioinformatics analysis of causal relationships of pathogenic and non-pathogenic gene sets affected by transcript level changes of the specific gene.

## 4 Inflammatory mediators and their sources

### 4.1 Cyto/chemokines

**Figure 1:**
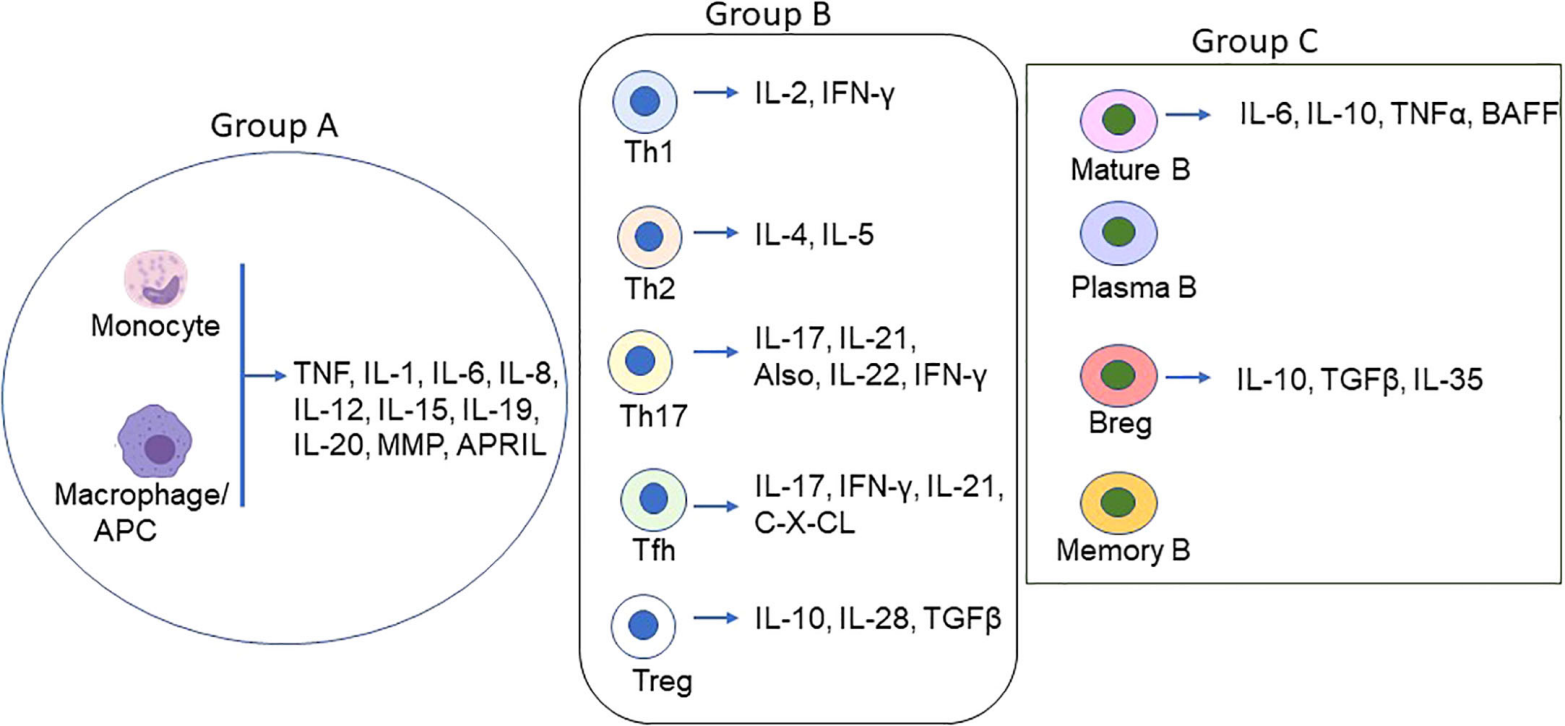
Schematic of Proinflammatory/inflammatory cytokines produced by main immune cell types in MG. Various cell types secret proinflammatory, inflammatory and inflammatory cytokines in addition to the groups (group A = monocytes/macrophages/DCs; group B = T lymphocytes, and group C = B lymphocytes) depicted above. The coordinated function of the cyto/chemokines prime activates DCs and AChR-specific Th/B cells and the ultimate differentiation of antigen-specific B cells to autoantibody-producing plasma cells and memory plasma B cells.

Cyto/chemokines are the small peptides produced virtually by all cells but primarily by myeloid cells in response to stimuli (**Figure 1**). They are critical modulators of inflammation and act as potent activators or inhibitors of immune responses (**Table 1**).

**Table 1:** Cyto/Chemokines: potent contributors of MG pathogenesis.

Cyto/Chemokines	Main cellular sources	Regulation		MG associated major function	Treatment Responses	Ref.
		Up	Down			
TNF	Monocyte, Th1, Mφ	**↑**		Proinflammatory	Conditional	(15, 33–37)
IL-1β	Macrophages	**↑**		Proinflammatory	Unknown	(38)
IL-4	Th2, mast cells, eosinophils, basophils		**↓**	Pleiotropic antiinflammatory cytokine	Present	(38, 39)
IL-6/IL-6R	Monocyte,	**↑**		Proinflammatory	Present. Satralizumab (Active)	(6, 13, 40)
IL-8	Monocyte, Mφ	**↑**		Inflammatory chemokine	None	(6)
IL-10	Treg, Th2, monocytes, activated B cell subsets		**↓**	Antiinflammatory	-	(6)
IL-17A/C	Th17 cells, strong	**↑**		Proinflammatory	Inconclusive	(41, 42)
IL-12	Monocyte, Mφ	**↑**		Proinflammatory	Unknown	(38, 39)
IL-15	Monocyte, Mφ, DCs	**↑**		Proinflammatory	Unknown	(38)
IL-19	B cells, monocytes	**↑**		Induces IL6 and TNF expression; helps in Th2 functions	Present	(38)
IL-20	Monocyte, DCs, granulocytes	**↑**		Proinflammatory	Present	(38)
IL-22	Th17, Mφ	**↑**		Both pro- and antiinflammatory; skin inflammation	Present	(43)
IL-28 A	Tregs, DCs		**↓**	Suppress proinflammatory cytokines	Present	(38)
IL-35	Tregs, B regs		**↓**	Suppress effector B and T cells	Present	(38)
eotaxin	Eotaxin 1/ccl11: Produced by TNF-stimulated monocytes IFN**γ**-stimulated endothelial cells	**↑**		Chemoattractant for Th2 cells, eosinophils, and basophils.	Unknown	(38)
APRIL	Mφ, DCs, eosinophils, and neutrophils.	**↑**		Costimulator of B and T cell proliferation	Present	(38)
MMP	Mφ, neutrophils, T cells	**↑**		Wound healing	Unknown	(6)
TGFα	T reg cells	**↑**		Suppress CD8 cells,	-	(6, 44)
EN-RAGE	Monocytes, T cells, endothelial cells	**↑**		Activates inflammatory cascades	Unknown	(6)
BDNF	T cells, B cells, monocytes	**↑**		Promote inflammation	Unknown	(6)
VEGF	Mφ	**↑**		Proinflammatory	Unknown	(38)
CCL19	T cells, DCs	**↑↓**		Dual role (Pro- or antiinflammatory)	-	(6, 7)
C-X-CL	Tfh cells,	**↑**		Proinflammatory	Unknown	(6, 7, 45)
MIP-1(α, β)/CCL3	Monocytes, T/B cells, DCs, NK cells	**↑**		Recruit inflammatory cells	Unknown	(6)
IFN	Th1	**↑**		Pleiotropic function; regulate Th17 cells, modulate DC antigen presentation	Not present	(23, 33, 46–48)
*BAFF*	B cells, T cells.	**↑**		Survival and maturation of B cells	Present	(7, 49)
Hmg-1	Ubiquitous, all cells	**↑**		Promote inflammation	Unknown	(50, 51)

IL, interleukin; APRIL, A proliferation-inducing ligand; MMP, Matrix metalloproteinases; TGF, transforming growth factor; EN-RAGE, Extracellular newly identified receptor for advanced glycation end products binding protein; BDNF, Brain-derived neurotrophic factor. Up arrow, upregulated; down arrow, downregulated.

#### 4.1.1 Monocytes/Macrophages

Cytokine producing role of nearly all immune cells is known; however, FcR-expressing monocytes/macrophages are the primary sources of the proinflammatory/inflammatory cytokines and chemokines, including mainly TNF, IL-1, IL-6, IL-8, and IL-12 causing histopathological injuries in target tissues, autoantigen expressions, and autoantibody production in MG and other autoimmune diseases (38, 52). IL-6 is a pleiotropic cytokine, released mainly from monocytes and macrophages but also from other cells, including B cells, muscle, and epithelial cells. High levels of IL-6 are associated with the pathogenesis of many diseases. Mice with an acquired or genetic deficiency of IL-6 are resistant to MG, and anti-IL6 antibodies reduced autoantibody levels and disease symptoms in a rat model of EAMG (13, 53). Following binding with its receptor, IL6 receptor (IL6-R), CD26, or soluble IL6-R, the complex binds to CD136, which dimerizes and activates intracellular kinases. The role of Granulocyte-macrophage colony-stimulating factor (GM-CSF), produced by macrophages, endothelium, and other cells, as pro- or anti-inflammatory cytokine is controversial.

Another less-studied intracellular DNA binding protein with a proinflammatory cytokine-like role in MG pathogenesis is the High mobility group box 1 (HMGB1) (50). Extracellular HMGB1 secreted passively from monocytes and other cell types bind to TLR4 of monocytes and macrophages and induce ehnanced production of TNF-α, IL-1, IL-6, IL-8, macrophage inflammatory protein (MIP), C-reactive protein (CRP) and others to amplify inflammatory responses.

#### 4.1.2 Neutrophils

Although the role of neutrophils has not been studied well for MG pathogenesis, they play a significant damaging role in many autoimmune diseases, such as anti-glomerular basement membrane nephritis and lupus nephritis (54).

#### 4.1.3 T lymphocytes

T lymphocytes or T cells play a role in the autoantibody synthesis of B cells through the secretion of various cytokines following stimulation with AChR in MG. Of T cells, T-helper cells 2 (Th2) secret IL-4 and IL-5 that helps in T and B cell growth. Th1 cells produce IL-2 and IFN-γ. T helper 17 (Th17) cells secret mostly IL-17, IL-21, and IL-22 but also IL-23 and IFN-γ (55), and their frequency increases with MG disease severity. Evidence demonstrating stimulus-mediated polarization of Th cells from one subtype to another, such as Th1 to Th17 cells and vice versa (56), might play a crucial role in the pathogenesis of MG.

T follicular helper (Tfh) cells are a subset of CD4 cells (CD4+CXCR5+PD1+) that also secrete IL-17 and IFN-γ. An increase in Tfh cells in correlation with increased numbers of plasma cells and AChR-specific autoantibody titer has been reported from generalized MG patients (57). Tfh cell frequencies were found to decrease after treatment. The-secreted cytokine, IL-21, has been reported to be elevated in MG patients with higher QMG scores and anti-AChR antibody (12). T regulatory cells (Tregs) are indispensable for maintaining peripheral tolerance through suppressing CD4 cells. Reduced expressions of Treg markers (e.g., FoxP3 and IL-10) cause Treg dysfunction and are reported to be associated with increased levels of proinflammatory cytokines, IL-6, IL-17 in MG patients, and IFN-γ (46, 58).

#### 4.1.4 B lymphocytes

A crucial pathogenic role of B cells in autoantibody-mediated autoimmune disease is known. In addition to producing anti-AChR autoantibody, B cells play an important role in the presentation of autoantigen, secretion of proinflammatory cytokines, interaction with T cells through expressing costimulatory molecules, and formation of ectopic germinal centers in autoimmune diseases. Mature B cells are mainly located in the secondary lymphoid organs but also in peripheral circulation and tissues (tissue-resident B cells). Various cell surface markers, e.g., CD269 and CD138/Syndecan-1 on plasma cells, CD19, CD20, CD21, CD268, and CD79b on mature B cells, and CD27 on memory B cells are present or upregulated on B cell surface during disease progression and are useful targets for current B cell depletion therapies for MG (7). In contrast to T reg cells, B regulatory cells (Bregs) cells lack specific surface markers and are recognized by the secretion of IL-10. Reportedly, CD19+IL-10+ (more precisely, CD19+CD5+CD1d+ and CD19+CD24+CD38+ subsets) Breg cell numbers are significantly lower in peripheral blood and thymus of MG patients compared to healthy controls, and that correlates with disease severity (59–61).

#### 4.1.5 Dendritic cells (DCs)

Dendritic cells are differentiated from monocytes and are the professional antigen-presenting cells (APCs) that promote B- and T-cell activations and differentiation. Percentages of plasmacytoid DCs (pDCs) are known to increase significantly over myeloid DCs (another DC subset) in naïve MG patients (62) compared to healthy controls. Activated DCs migrate to secondary lymphoid organs and secret IL-12, IL-6, TNF- and many chemokines, and CD303+ pDCs produce high levels of IFN-α.

### 4.2 Chemokines

Proteins belonging to the CC and CXC family of chemokine ligands and receptors, secreted by peripheral blood mononuclear cells, lymph node cells, macrophages, and thymic GCs also contribute to inflammation in MG (7, 45). Based on *in silico* analyses, potential therapeutic chemokine targets for MG could be CXCR2, CXCR3, CXCL1, CXCl3, CCL, CCL19, and CCL20 (7).

## 5 Inflammation, immune complexes, and fragment crystallizable receptor

Fc receptors (FCRs) of some hematopoietic cells that bind to the Fc domain of many Immunoglobulin (Ig) subclasses play an important role in the pathogenesis of MG and other autoimmune diseases. Classical FcRs are activating Fcγ(gamma)Rs (CD64, CD32a, CD16a/b) and inhibitory FcγRs (CD32b), and nonclassical FcR includes only FcRn. Autoantibody bound to an autoantigen, i.e., immune complex (IC), can lead to inflammation through the aggregation of FCRs (45). Targeting FcR is a novel therapeutic approach for some autoimmune diseases. In a mouse model of MG, recombinant Fc multimers (IgG Fc analogue) have been shown to effectively block complement activation by a monoclonal antibody against FcγR. Recombinant soluble FcγRs have not been tested in MG. A clinical trial is underway evaluating a humanized anti-FcγRIIA antibody (VIB 9600) that was recently shown to be effective in inhibiting IC-induced production of type 1 IFN by plasmacytoid dendritic cells and TNF and IL-6 by monocytes (18). The antibody has not been tested for MG. Several monoclonal antibodies against an IgG recycling receptor, neonatal Fc Receptor (FcRn) (63), are currently in MG clinical trial, and some show promise. FcRn blockade has been shown to decrease circulating IgG levels in mice and humans (64) as opposed to the finding of a group showing that despite reducing inflammation, FcRn blockade did not alter the levels of circulating autoantibody in the rheumatoid arthritis (RA) model. A decrease in inflammatory disease burden, as determined in this study by clinical scoring of histopathologic arthritic inflammation and animal mobility, affirms that FcRn is also directly associated with IgG IC-associated inflammation (65). Molecular mechanisms underlying this correlation or association, however, require more research.

Of various FcRn mAbs, Efgartigimod (ARGX-113; Argenx, Breda, the Netherlands) or VYVGART™ was recently approved by U.S. Food and Drug Administration (FDA) for the treatment of AChR seropositive generalized myasthenia gravis (gMG) in adult patients.

## 6 Therapeutic potentials for targeting inflammation in MG

Most of the current targeted therapy for MG showed good success in clinical trials. Monotherapies targeting various inflammatory mediators (such as IL-6 or IL6-receptors) are currently active in clinical trials. Tocilizumab (TCZ; RoActemra^®^ or Actemra^®^; Roche, Basel, Switzerland), also known as atlizumab, is a recombinant humanized mAb against the membrane bound or soluble IL6 receptor. Tocilizumab treatment has shown clinical efficacy in MG patients refractory to rituximab and other diseases, including rheumatoid arthritis (RA), juvenile idiopathic arthritis, Castleman’s disease, and Crohn’s disease (33, 40). However, some solo anti-inflammatory therapeutic treatments did not produce striking outcomes. For example, etanercept (a TNF-α antagonist decoy receptor) was beneficial for patients with low plasma levels of IL6 and interferon (IFN)γ (34), but it exacerbated MG in a patient with RA (35) and also reactivated tuberculosis (36, 37). Other anti-IL6 antibodies (e.g., sarilumab, sirukumab, and siltuximab were tested in treating RA or other diseases but not in MG (66). Humanized anti-IFNα mAbs (Rontalizumab, developed by Genentech (San Francisco, CA, USA) and anifrolumab, developed by Medimmune (Gaithersburg, MD, USA) produced contradictory results in MG or mice model of MG (47, 48), and therefore no clinical trial in MG was conducted.

Investigations are also ongoing to assess novel treatments for MG. Strategies to design a targeted therapy paired with novel molecules targeting inflammation can be beneficial.

Under the influence of local cytokines, CD4+ T cells, through expressions of the lineage-specific transcription factor, can differentiate into Th1, Th2, Th17, or Tregs (e.g., T-bet or TBX21 for Th1, GATA-3 for Th2, Fos-like antigen 2 or Fosl2 for Th17, and FoxP3 for Tregs) (44). Such phenotypic and functional plasticity of Th cells have not been studied in MG, and thorough future investigations in this line remain to be seen. Strategies to manipulate properties of immune cell plasticity through designing appropriate biologic, inhibitor, or microRNA, and thus increasing or equilibrating the number of Tregs and reducing Th1/Th17 type inflammatory cytokines (67) could be a potential therapeutic strategy for MG. One recent study, however, failed to find any observed clinical benefit of using an IL-17-specific monoclonal antibody (Secukinumab) in patients with MuSK-MG (41). Treatment nonresponse to the IL-17-directed therapy could be due to prior exposure to immunosuppressive therapies in the patients. Further, it has recently been shown that not all Th17 cells producing IL-17 are pathogenic or induce autoimmune responses (68). More research in this line is necessary to dissect the mechanism of action of IL-17 antagonists. Expansion of IL-10-producing B-regulatory (B-reg) cells through expressing inhibitory coreceptors such as CD22 (69–71) and suppressing B cell activation is another mechanism to investigate and exploit for developing future B cell-directed therapy.

## 7 Conclusions

In conclusion, both the basic- and clinical research data show evidence of a clear association of inflammation with MG. Utilizing new technologies and highly efficient tools for research and data collection, it is possible to conduct a more comprehensive mechanistic study about the role of inflammation in autoimmune development or modulation of the autoimmune response in preclinical models of MG and MG. The results will provide deeper insights to help design more precise and effective therapies. Future therapy approaches for MG may also assess the effectiveness of combination therapy targeting both components, autoantibody and inflammation, responsible for MG pathogenesis.

## Author contributions

The author reviewed the literature, contributed solely to the writing, and approved the manuscript for publication.
